# Wave propagation enhances extracellular signal strength in small excitable tissues

**DOI:** 10.1038/s41598-026-51791-6

**Published:** 2026-05-07

**Authors:** Karoline Horgmo Jæger, Aslak Tveito

**Affiliations:** Simula Research Laboratory, Oslo, Norway

**Keywords:** Biological techniques, Biotechnology, Cell biology, Engineering, Stem cells

## Abstract

Human induced pluripotent stem cell-derived cardiomyocytes (hiPSC-CMs) offer new opportunities to study cardiac dynamics and drug responses. High-throughput platforms often rely on extracellular potential (EP) recordings, making signal strength critical for analysis. To examine how stimulation protocols shape EPs, we used a computational model of hiPSC-CMs cultured in a well geometry and compared extracellular recordings under uniform and localized stimulation. Uniform global stimulation activated all cells simultaneously, suppressed wave propagation, and produced only small extracellular signals despite robust transmembrane action potentials. Localized stimulation, by contrast, initiated traveling waves and generated strong, well-defined EPs. These findings show that the stimulation protocol directly determines EP strength in dish-based assays and have practical implications for the design and interpretation of high-throughput hiPSC-CM experiments and other in vitro electrophysiological measurements.

## Introduction

The development of cardiomyocytes derived from human-induced pluripotent stem cells (hiPSC-CMs) has enabled in vitro studies of human cardiac cell behavior^1–3^. These cells are increasingly used to assess disease mechanisms and drug-induced effects^4–7^. To support large-scale testing, hiPSC-CMs are commonly cultured in multiwell platforms or integrated into microphysiological systems (MPSs)^8–10^. These platforms often rely on extracellular potential (EP) recordings to infer electrophysiological activity^11–13^. Here we show that the interpretability of EPs depends critically on the stimulation strategy. In particular, we point out that the common approach (see, e.g., ref. ^14^) of simultaneous stimulation of all cells in the dish, produces the weakest EP signal and should therefore be avoided.

For a uniform cable, it was shown in ref. ^15^ that the EP is determined by the second spatial derivative of the transmembrane potential; in particular, if the potential is completely uniform, the EP vanishes. This observation was extended in ref. ^16^, where it was demonstrated across several physiological systems that autonomous, fully homogeneous cells generate no measurable EP, even during full action potentials. Specifically, the result was shown for sinoatrial node pacemaker tissue, for cultures of hiPSC-CMs in heart-on-chip configurations, for pancreatic $\beta$-cell islets, and for cerebellar Purkinje neurons. In all cases, extracellular silence persists as long as the cells are homogeneous and synchronous. To analyze such systems using extracellular recordings, spatial voltage gradients must be introduced—typically by applying external stimulation. These gradients then give rise to measurable EPs. Here, we examine how different stimulation protocols shape the spatial activation pattern and thereby influence the quality of the extracellular signal.

Using a computational model applied to two different geometries, we compare four conditions: (i) no stimulation, where cells beat autonomously; (ii) weak uniform global stimulation; (iii) strong uniform global stimulation; and (iv) localized stimulation to initiate wave propagation. Our computations show that conditions (ii)–(iii) yield weak EPs, whereas condition (iv) produces strong, well-defined signals due to the traveling excitation wave. Also (i) may yield strong signals if the variability of cell properties are sufficiently strong, whereas (ii) and (iii) always yield weak signals. This demonstrates that the stimulation protocol plays a critical role in generating measurable extracellular signals.

## Methods

### Computational model

**Fig. 1:**
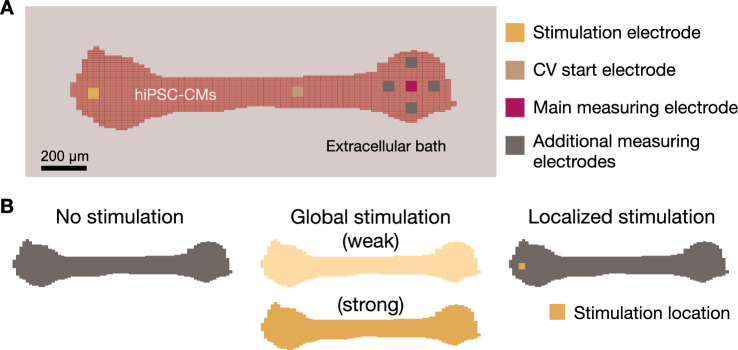
**A**: Default model geometry. hiPSC-CMs are arranged in a dogbone-like geometry in a single well (see Chip C1 in ref. ^18^). Each cell has a length of 25 $\mu$m and a width of 8 $\mu$m. The location of one stimulation electrode and six measuring electrodes are indicated. Note that hiPSC-CMs are also located at the location of the electrodes. **B**: Stimulation protocols. We consider four different stimulation protocols: (i) no stimulation (spontaneous action potential firing), (ii) weak global optical stimulation applied in the entire cell collection, (iii) same as (ii) but stronger stimulation, and (iv) localized extracellular stimulation through an electrode.

We perform simulations of a collection of hiPSC-CMs using the Kirchhoff network model (KNM)^17^. The default model parameters, cell collection geometry, and the model for the single-cell dynamics are adapted from Chip C1 in ref. ^18^, with membrane dynamics based on ref. ^19^. The cell collection consists of a two dimensional sheet of 1,637 hiPSC-CMs connected to their neighbors by a default gap junction strength of $G_g=1.7$ nS. The cell collection is surrounded by an extracellular bath, and a Dirichlet boundary condition of the form $u_e=0$ mV is applied on the outer boundary of this bath. In Fig. 1A, the geometry of the cell collection is illustrated. In addition, the location of stimulation and measurement electrodes are indicated.

### Stimulation approaches

In Fig. 1B, we illustrate the stimulation approaches considered in this study. In the case of no stimulation, the cells beat spontaneously at a rate determined by their intrinsic pacemaker properties. The global stimulation is set up to mimic global optical pacing of the cells, represented by the activation of a negative 5 ms long transmembrane current. We consider two different strengths of this light activated current, one weak current of 3 $\mu$A/cm$^2$, which is barely enough to initiate an action potential and one strong current of 30 $\mu$A/cm$^2$, which is more than enough to initiate action potentials. For the localized stimulation, a 5 ms long constant current of 0.02 $\mu$A is injected into the extracellular space through a stimulation electrode. All stimulation is applied at 1 Hz.

### Conduction velocity

In our simulations, we perform simulations using different values of the gap junction strength, $G_g$. To relate the value of $G_g$ to tissue conduction, we compute the conduction velocity for the case of localized stimulation. The conduction velocity is defined as the distance between the so-called “CV start electrode” and the main measuring electrode displayed in Fig. 1A divided by the time between the peaks in the EP recorded in these two electrodes.

**Fig. 2:**
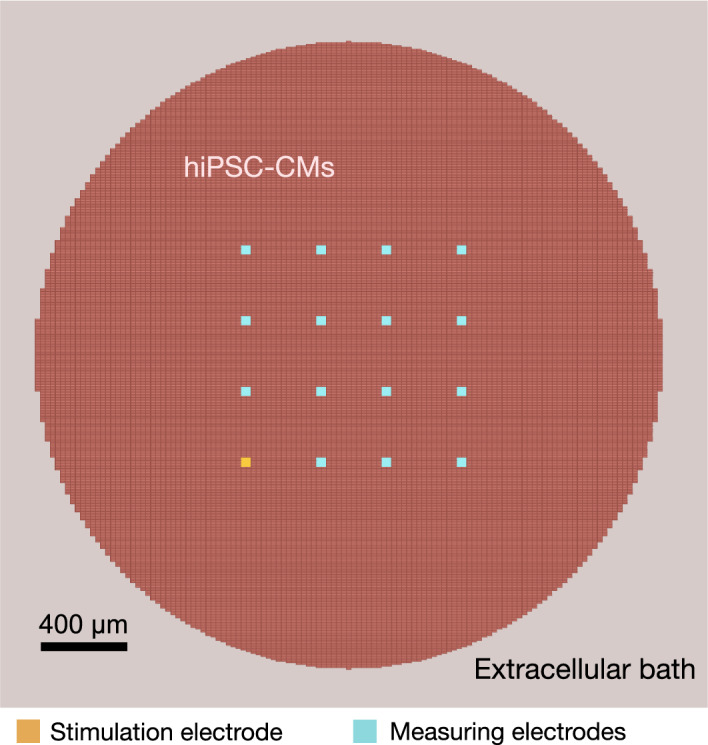
Circular MEA geometry used in some simulations. hiPSC-CMs are arranged in a circular geometry with radius 1.56 mm. Each cell has a length of 25 $\mu$m and a width of 8 $\mu$m. The location of one stimulation electrode and 15 measuring electrodes are indicated. The electrode width is 50 $\mu$m and the distance between electrodes is 350 $\mu$m. Localized stimulation is applied in the stimulation electrode, and we plot the extracellular potential (EP) and membrane potential measured in the 15 measurement electrodes for all stimulation types. The four different applied stimulations are as described in Fig. 1B.

### Circular MEA geometry

In addition to the default hiPSC-CM chip geometry described above and in Fig. 1, we also perform a set of simulations using a geometry inspired by commercially available microelectrode array (MEA) platforms. In particular, we use a circular geometry based on the BioCircuit MEA 24 plate from Axion BioSystems, used in, e.g., ref. ^20^. The plated area is circular with a radius of 1.56 mm, corresponding to an area of 7.65 mm^2^, and it contains 38,239 cells and 16 electrodes, as illustrated in Fig. 2. We apply the same model parameters, stimulation approaches and simulation setup as for the dogbone-like geometry.

### Numerical methods

The KNM system is solved using a standard first order operator splitting scheme, splitting the non-linear single-cell dynamics from the linear part of the KNM system (see, e.g., refs. ^17,21,22^) with a global time step of $\Delta t = 0.1$ ms and a local time step of $\Delta t = 0.0005$ ms for the forward Euler scheme used for the non-linear single-cell dynamics.

## Results

### Identical cells

**Fig. 3:**
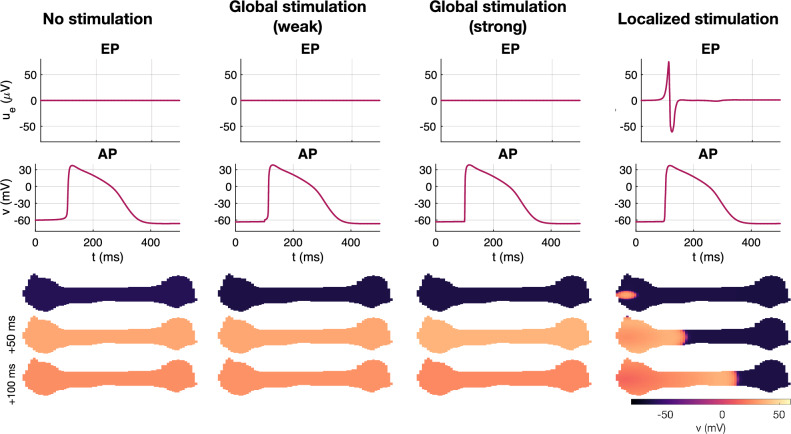
Identical cells. Simulation of a collection identical hiPSC-CMs for four different stimulation protocols. The upper panel displays the extracellular potential measured in the main measurement electrode illustrated in Fig. 1A. The extracellular potential is zero in all cases except for localized stimulation. The next panel shows the membrane potential recorded at the location of the same electrode. All stimulation protocols display clear and similar action potential (AP) firing. In the lower panel, snapshots of the membrane potential are displayed for three points in time during AP firing. For the localized stimulation, an excitation wave is generated with a conduction velocity of 1.0 cm/s, whereas for the remaining stimulation protocols, the cells fire uniformly across the cell collection.

We begin by considering a collection of identical, autonomously beating hiPSC-CMs using the computational model derived in refs. ^17,18^. Figure 3 shows that stimulation cases (i), (ii) and (iii) generate no EPs even though all cases exhibit full transmembrane action potentials. In contrast, localized stimulation (iv) induces a traveling wave, leading to strong extracellular signals.

### Varying cell properties

**Fig. 4:**
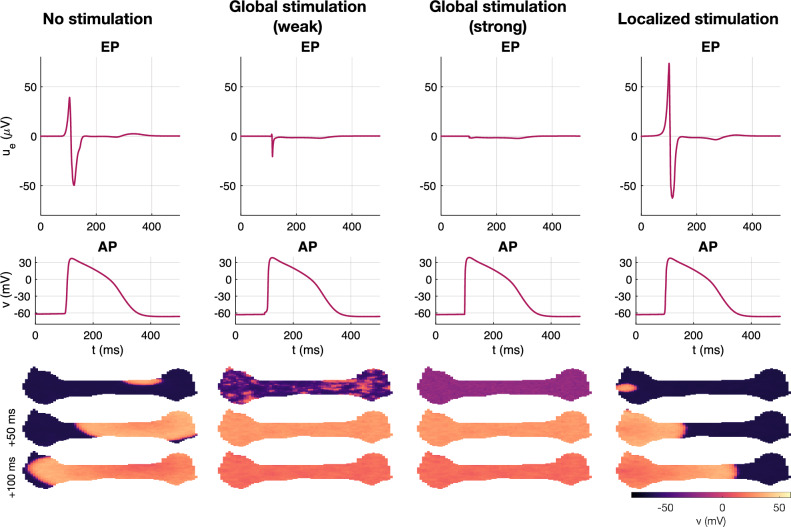
Varying cell properties. Simulations like in Fig. 3, except that cell-to-cell variation is introduced in the conductances of the membrane currents (20% standard deviation). In this case, there are spatial gradients in the membrane potential for all stimulation protocols. Consequently, EPs are present in all cases, but wave initiation continues to yield the strongest signal.

Figure 4 shows the effect of introducing cell-to-cell variation in the ion channel conductances. For each cell, a separate random scaling factor is drawn for each ionic current and used to modify the corresponding maximum conductance. The scaling factors are drawn from a normal distribution with expectation equal to the default conductance value and standard deviation of 20% of the default conductance value.

In Fig. 4, we observe that cell-to-cell heterogeneity enhances EPs in both the no stimulation (autonomous) case and in the global stimulation cases, but localized stimulation still produces the strongest and most reliable signal, driven by the wavefront. In the no stimulation case, the heterogeneous cell properties lead to action potential firing occurring at a location in the upper right part of the cell collection before the remaining cells fire, resulting in a traveling excitation wave similar to that generated in the localized stimulation case. For a weak global stimulation, certain spatial differences are present during depolarization, resulting in an extracellular signal that is visible, yet weaker than for the no stimulation and localized stimulation cases. For the strong global stimulation, the stimulation current evens out these spatial differences to a large extent, and the resulting extracellular signal is very weak.

### Improved gap junction coupling

**Fig. 5:**
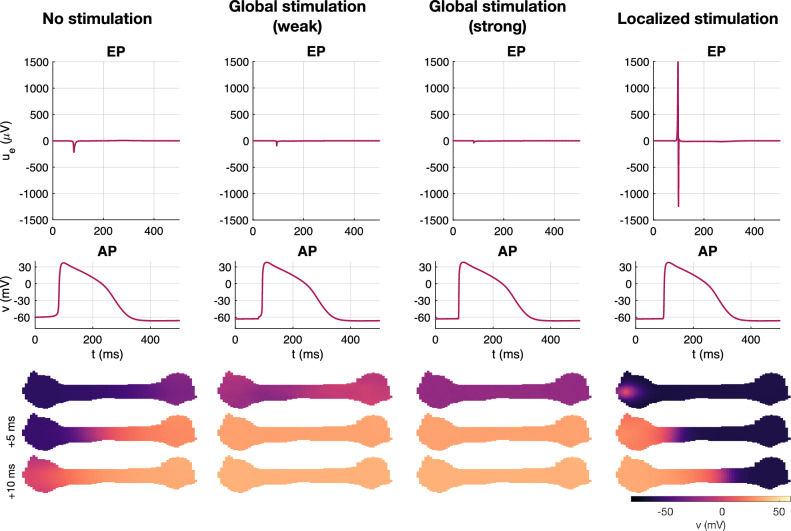
Improved gap junction coupling. Simulations like in Fig. 3 except that the gap junction coupling strength is increased by a factor of 100, to $G_g = 170$ nS, and that there is 20% cell-to-cell variation in ion channel conductances. The increased gap junction coupling greatly enhances the EP signal strength for a localized stimulation.

Due to the immature nature of hiPSC-CMs, gap junction coupling is not fully developed and may vary across different hiPSC-CM cell collections. For example, in ref. ^18^, the conductance of the gap junctions connecting neighboring cells was found to vary between $G_g=1.7$ nS and $G_g=180$ nS for different cell collections. Figure 5 shows the effect of increasing the gap junction strength by a factor of 100 to $G_g=170$ nS. This increases the conduction velocity of the tissue from 1.0 to 9.8 cm/s. We still consider 20% variation in cell properties. We observe that when the gap junction conductance is increased, the extracellular signal strength for the localized stimulation is considerably improved and consistently stronger compared to the other stimulation approaches.

### Summary of perturbed properties

**Table 1:** The four righmost columns provide the maximum amplitude of the extracellular potential (in $\mu$V) over all five measurement electrodes illustrated in Fig. 1. The cell-to-cell variation is represented by drawing ion channel conductances from a normal distribution with standard deviation of 0%, 5%, or 20%, and $G_g$ represents the gap junction coupling strength between neighboring cells. The conduction velocity (CV) is computed for the localized stimulation case as described in "Section Conduction velocity".

Cell variation (%)	$G_g$ (nS)	CV (cm/s)	None	Global (weak)	Global (strong)	Localized
0	1.7	1.0	0.0	0.0	0.0	74.8
	17	3.3	0.0	0.0	0.0	468.8
	170	9.8	0.0	0.0	0.0	1597.9
5	1.7	1.0	48.9	15.7	0.8	74.8
	17	3.3	54.7	41.7	7.0	468.7
	170	9.8	67.3	41.3	20.9	1607.2
20	1.7	1.0	49.7	35.0	3.3	73.7
	17	3.3	144.0	140.2	28.0	465.3
	170	9.8	232.9	176.9	83.5	1621.9

In Table 1, we have summarized how the maximum amplitude of the EP depends on different choices of the gap junction coupling strength and the degree of cell-to-cell variation. For all choices, the localized stimulated simulations yield the strongest EP signal. For any choice of gap junction coupling strength, 0% cell variation yields zero EP for the no stimulation and global stimulation cases. As cell variation is increased, the EP strength for the no stimulation case and the global stimulation cases increases, but the localized stimulation yields the strongest EPs in all cases and in particular for strong gap junction coupling. In addition, the no stimulation case consistently give rise to stronger EPs than global stimulation, and a strong global stimulation results in the weakest EP signals of the considered approaches.

### Circular MEA geometry

**Fig. 6:**
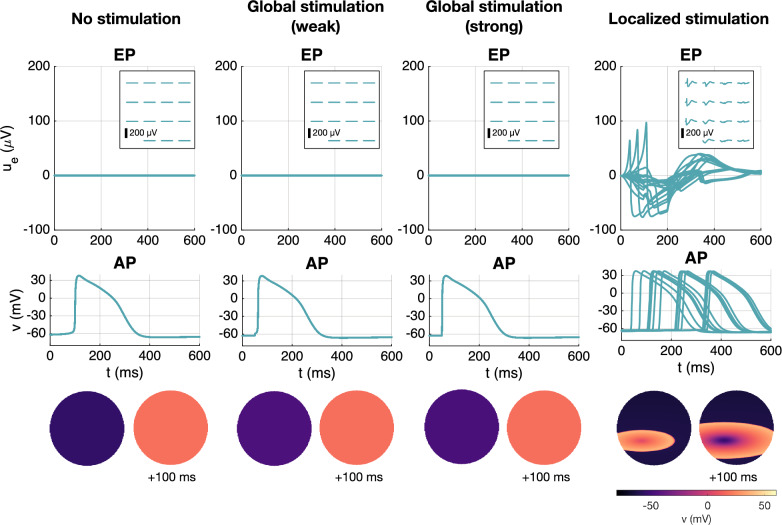
Identical cells in a circular MEA geometry. Simulations like in Fig. 3, except that we use a circular MEA geometry, as illustrated in Fig. 2. The upper panel shows the extracellular potential (EP) recorded in each measuring electrode and the insets show the location of the individual EP traces in the MEA setup. The second panel shows the membrane potential recorded in the measuring electrodes. All stimulation protocols display clear and similar action potential (AP) firing. In the lower panel, snapshots of the membrane potential are displayed for two points in time during AP firing. Like in Fig. 3, the cells fire uniformly across the collection for all stimulation protocols except for the localized stimulation.

**Fig. 7:**
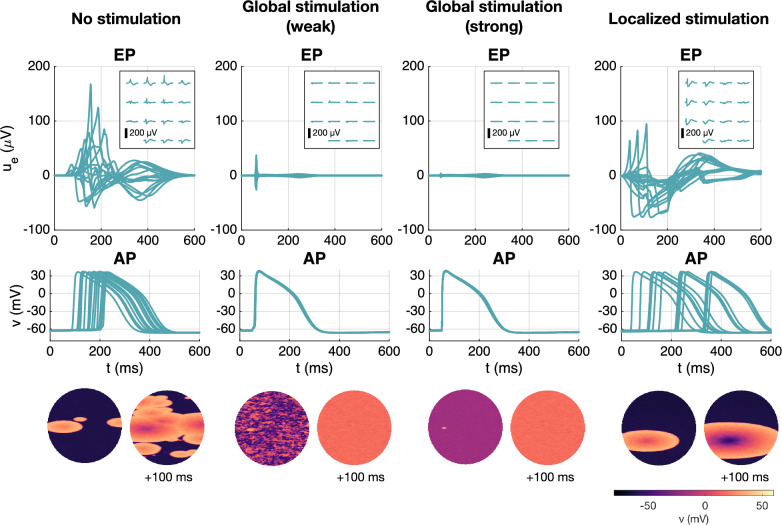
Varying cell properties in a circular MEA geometry. Simulations like in Fig. 6, except that cell-to-cell variation is introduced in the conductances of the membrane currents (20% standard deviation). Again, the EP signals are strongest for localized or no stimulation, weaker for a weak global stimulation, and weakest for a strong global stimulation.

**Fig. 8:**
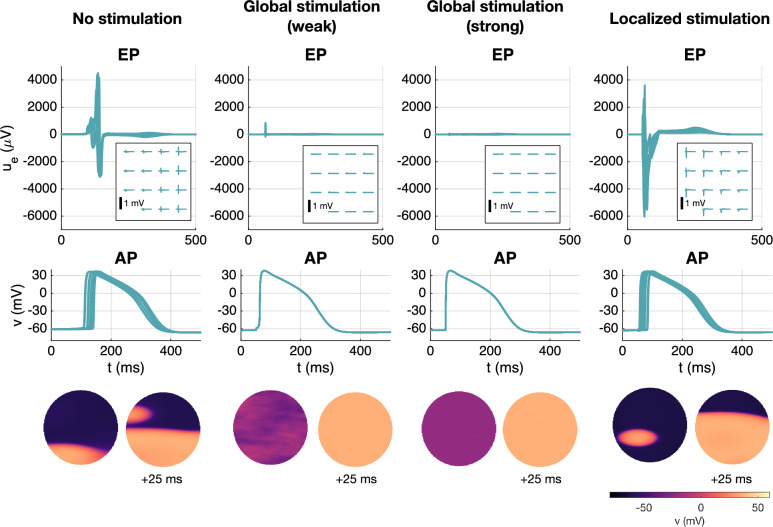
Improved gap junction coupling in a circular MEA geometry. Simulations like in Fig. 6, except that the gap junction coupling strength is increased by a factor of 100, to $G_g = 170$ nS, and that there is 20% cell-to-cell variation in ion channel conductances. The improved gap junction coupling greatly enhances the EP signal strength for the no stimulation and localized stimulation cases.

In a final set of examples, we consider a geometry inspired by commercially available MEA platforms. In particular, we use a circular geometry motivated by the BioCircuit MEA 24 plate from Axion BioSystems, used in studies such as ref. ^20^. The geometry is circular with radius 1.56 mm, corresponding to a plated area of 7.65 mm^2^, as illustrated in Fig. 2. We perform KNM simulations similar to those displayed in Figs. 3, 4, 5. As in the previous examples, we consider no stimulation, weak global stimulation, strong global stimulation, and localized stimulation. The results are presented in Figs. 6, 7 and 8. We observe that the conclusions are qualitatively the same as above: strong global stimulation gives rise to very weak EP signals, whereas localized stimulation (or no stimulation) produces the strongest signals.

## Discussion

The use of field potentials to assess drug-induced changes in hiPSC-CMs is well established^4,5,7,23–28^. Our results indicate that when analyses rely on extracellular potential (EP) recordings, the stimulation protocol plays a critical role in shaping signal strength. The clearest signals arise when stimulation initiates a traveling wave through the tissue. Autonomous activity without external stimulation can also yield measurable EPs, but uniform global stimulation consistently produces weaker signals. This effect is especially pronounced when strong global stimuli are used (see Table 1). Thus, if global stimulation must be used, the strength should be kept as low as possible while still reliably triggering action potentials, since stronger stimulation tends to suppress spatial voltage gradients and reduce EP amplitude. This effect is observed both in the dogbone geometry shown in Fig. 1 and in the circular geometry mimicking commercially available MEAs shown in Fig. 2.

When localized stimulation is applied, it must also be carefully tuned to avoid directly influencing the measurements; the observed signal should arise from the activity of the hiPSC-CMs, not from the input at the electrode. This is why the measurement electrodes are placed at the far end of the tissue in Fig. 1. Furthermore, if the cells are too weakly coupled for supporting a traveling excitation wave, localized stimulation will fail to generate a wave, and relying on intrinsic beating is likely the best option.

Stimulation that generates an excitation wave has the additional advantage that it permits measurement of the conduction velocity, which may provide an independent readout of sodium current strength and gap junction coupling^18,26,27^. In engineered cardiac tissues, conduction velocity is also commonly reported as a functional measure of electrical maturation, see, e.g., refs. ^29,30^. Reliable EP recordings may therefore provide information beyond signal amplitude alone. In Table 1, we observe that conduction velocity increases as the gap junction coupling is strengthened, consistent with the general observation that improved gap junction coupling is associated with faster propagation.

The situation depicted in Figs. 3 and 6 are idealized in the sense that all cells are assumed to be identical. This assumption is unrealistic, and in Figs. 4 and 7 we therefore consider the case of varying cellular properties. In that case, EPs are generated even under uniform stimulation, but the signals remain very weak when global stimulation is applied. Strong signals are achieved both when no stimulation is used and when localized stimulation is applied. We also note that other sources of inhomogeneity may be present, including non-uniform global stimulation, but we expect such effects to be qualitatively similar.

Variability in drug response detectability across hiPSC-CM platforms (Axion, Ncardia, Fujifilm, Pluricyte) has been noted in ref. ^31^, particularly in settings where analysis relies on spontaneous activity. Our results suggest that part of this variability may arise from differences in how activation spreads across the tissue, not just from differences in cellular properties. In ref. ^32^, extracellular signals remained below 2 $\mu$V despite full action potentials, especially when activation was spatially uniform. This supports the idea that spatial voltage gradients—not just cellular excitability—are required for generating robust EPs. Enhanced spike amplitudes observed during electrical pacing in MEA studies^20^—often doubling the signal compared to spontaneous activity—are consistent with this interpretation. These findings may also help explain why uniform optogenetic stimulation sometimes fails to reveal subtle pharmacological effects^14^.

A related issue, beyond the scope of the present note, is the inverse problem of attributing changes in recorded EPs to specific underlying electrophysiological mechanisms. In general, such an interpretation is not expected to be unique, since different combinations of altered ionic currents and intercellular coupling may give rise to similar changes in recorded electrical signals^33–35^. This observation does not weaken the main point of the present study. On the contrary, it emphasizes that if the stimulation protocol suppresses the EP signal itself, the interpretation of drug-induced or condition-dependent changes becomes even more difficult. Stimulation protocols that generate a propagating wave therefore provide a better basis for subsequent analysis of EP recordings.

It should be noted that the importance of strong EP signals depends on the intended use of the recordings. For timing-based biomarkers such as beat rate or field potential duration, a weak but clearly detectable signal may be sufficient. However, if the aim is to extract more detailed electrophysiological information from extracellular recordings, signal quality becomes considerably more important. For example, EPs have been analyzed to infer information about the location and timing of underlying currents^36^, and inverse approaches have been proposed in which underlying electrical activity is inferred from multielectrode recordings^37^. Such applications clearly benefit from signals that are as accurate and robust as possible.

## Conclusion

The simulations of this report confirm that stimulation protocols have a strong influence on the strength of extracellular potentials (EPs) in small in vitro tissues. Stimulation that initiates a traveling wave produces spatial voltage gradients and robust EPs, while uniform global stimulation—despite its common use—yields much weaker signals. These results are directly relevant to the design of protocols for extracting electrophysiological biomarkers from hiPSC-derived cardiomyocytes.

## Data Availability

The data generated during this study are included in the article.

## Declarations

### Code availability statement

The code used to run the simulations of this manuscript is publicly available at Zenodo: https://doi.org/10.5281/zenodo.19849938.
